# Nutritional Modulation of the Immuno–Biomineral Interface: From Vascular Calcification to a Hypothesis-Generating Framework for Tumor-Associated Biomineralization

**DOI:** 10.3390/nu18193198

**Published:** 2026-09-28

**Authors:** Nishmitha N. Hegde, Jayalaxmi Shetty, Chaithra Lakshmi, Mithra N. Hegde

**Affiliations:** Department of Conservative Dentistry and Endodontics, Nitte (Deemed to Be University), AB Shetty Memorial Institute of Dental Sciences (ABSMIDS), Deralakatte, Mangalore 575018, India; drnishmitha.hegde@nitte.edu.in (N.N.H.); jayashetty21@gmail.com (J.S.); chaithra.25pbos104@student.nitte.edu.in (C.L.)

**Keywords:** Immuno–Biomineral Interface, biomineralization, vascular calcification, tumor calcification, extracellular vesicles, nutrition, magnesium, vitamin K_2_, polyphenols, omega-3 fatty acids, macrophages, calcium signaling, inflammation, cancer

## Abstract

Pathological biomineralization is increasingly recognized as a regulated biological process involving interactions among mineral metabolism, inflammation, cellular stress, extracellular vesicles (EVs), extracellular matrix (ECM) components, and mineral precursors. These processes are particularly well characterized in vascular calcification, where immune, metabolic, vesicular, and matrix-associated pathways contribute to ectopic mineral deposition. Tumor-associated mineralization is also documented in several malignancies, but its cellular origins, mineral composition, and biological significance remain substantially less defined. The Immuno–Biomineral Interface (IBI) is proposed here as a hypothesis-generating conceptual framework for integrating these interacting processes and examining potential mechanistic connections between established vascular calcification pathways and tumor-associated biomineralization. Nutritional status can modulateseveral biological processes implicated in pathological calcification, including mineral homeostasis, inflammatory signaling, oxidative stress, ECM remodeling, and EV biology. Magnesium, vitamin K_2_, omega-3 fatty acids, and selected polyphenols have been investigated in experimental and clinical contexts, although the strength and consistency of evidence vary according to nutrient, dose, biological system, and outcome. The strongest evidence for nutritional modulation of pathological calcification derives from vascular and renal contexts. These findings provide a mechanistic basis for considering nutrition as a potentially modifiable influence on IBI-associated processes, rather than as a single upstream determinant of pathological biomineralization. Most mechanistic evidence reviewed here derives from vascular calcification. Tumor microenvironments nevertheless exhibit processes also implicated in pathological calcification, including chronic inflammation, ECM remodeling, altered calcium and mineral handling, cellular stress, and EV-mediated communication. These observations identify potential points of mechanistic convergence but do not demonstrate that vascular and tumor-associated mineralization arise through equivalent pathways. Importantly, direct evidence that defined nutritional exposures modify the initiation, composition, maturation, or spatial distribution of tumor-associated mineral deposits remains scarce. The proposed nutrition–IBI–tumor biomineralization relationship should therefore be regarded as a testable, hypothesis-generating model rather than an established causal pathway. Asa narrative review, we integrate evidence from nutritional science, vascular biology, immunology, EV research, mineral metabolism, and cancer biology to critically distinguish established mechanisms from indirect evidence and hypothesis-generating extrapolations. We further highlight key knowledge gaps concerning nutrient-dependent regulation of immune responses, EV biology, matrix remodeling, and mineral formation within tumor microenvironments. Future studies combining defined nutritional interventions with spatial molecular profiling, EV characterization, high-resolution mineral characterization, and quantitative pathology will be required to determine whether mechanisms established in vascular calcification extend to tumor-associated biomineralization. The principal value of the IBI framework is therefore to organize existing evidence and define experimentally testable relationships between nutrition, pathological mineralization, and tumor biology while maintaining clear boundaries between established and unvalidated mechanisms.

## 1. Introduction

Pathological biomineralization is observed across cardiovascular disease, chronic kidney disease, inflammatory disorders, and cancer. Historically regarded as a passive consequence of tissue injury or calcium–phosphate precipitation, pathological calcification is now understood to involve regulated cellular, extracellular, and physicochemical processes. Extracellular vesicles (EVs), immune cells, extracellular matrix (ECM) components, mineral-associated proteins, and calcium–phosphate species can interact to influence mineral nucleation, crystal maturation, and tissue remodeling [1,2,3,4,5,6,7]. However, the relative contribution of these mechanisms varies considerably across tissues and disease states.

Among pathological calcification disorders, vascular calcification provides the most extensively characterized mechanistic model of ectopic biomineralization. Experimental and clinical studies have demonstrated that vascular mineralization involves osteogenic reprogramming of vascular cells, EV-mediated mineral nucleation, ECM remodeling, inflammatory signaling, oxidative stress, and disturbances in calcium–phosphate homeostasis [1,2,5,6,7]. These findings have established that vascular calcification is not simply an inert deposition of mineral but a biologically regulated process involving interactions between cellular phenotypes, matrix composition, mineral metabolism, and local inflammatory conditions.

Tumor-associated biomineralization represents a distinct but potentially informative context. Calcified deposits and microcalcifications have been described in several malignancies, including breast, thyroid, and ovarian tumors, where mineral composition, tissue architecture, cellular degeneration, ECM remodeling, and local inflammatory processes may contribute to mineral formation [8,9]. Nevertheless, the biological mechanisms underlying tumor-associated biomineralization remain substantially less defined than those governing vascular calcification. In particular, the extent to which immune cells, EVs, mineral-associated proteins, and altered calcium handling directly regulate mineral formation within tumors remains uncertain. Therefore, similarities between vascular and tumor microenvironments should be considered mechanistic parallels that generate testable hypotheses rather than evidence of equivalent biological processes.

Nutrition provides an important biological context in which these pathways may be regulated. Dietary factors influence systemic calcium and phosphate availability as well as endocrine regulators such as parathyroid hormone, fibroblast growth factor 23, and vitamin D. Nutritional status can also modify oxidative stress, immune-cell function, inflammatory signaling, ECM remodeling, and EV biology [10,11,12,13]. These effects provide several potential routes through which dietary factors could modify the tissue conditions that favor or restrain pathological mineralization. However, the strength of evidence differs substantially among nutrients and disease contexts, with much of the mechanistic evidence derived from vascular calcification, experimental models, or studies of mineral metabolism rather than direct nutritional intervention studies in patients with calcific disease.

Against this background, we propose the Immuno–Biomineral Interface (IBI) as a hypothesis-generating conceptual framework for integrating interactions among immune cells, EVs, ECM components, mineral-associated proteins, and developing mineral deposits. The proposed IBI does not represent an established anatomical or molecular structure. Rather, it describes a biological interface in which inflammatory signaling, immune-cell activity, matrix remodeling, vesicle-mediated communication, mineral metabolism, and physicochemical processes may converge to influence pathological biomineralization [5,7,14,15,16]. The framework is primarily informed by established observations from vascular calcification and related mineralizing disorders (Figure 1).

Within this framework, nutrition may function as an upstream modifier of multiple IBI-associated pathways, rather than as a single direct regulator of mineral deposition. Magnesium, vitamin K, omega-3 fatty acids, polyphenols, and other dietary factors have been investigated for their effects on mineral metabolism, oxidative stress, inflammation, cellular phenotype, and tissue remodeling. Nevertheless, these nutrients differ substantially in their mechanisms, bioavailability, experimental evidence, and clinical support. Moreover, evidence that nutritional modulation of these pathways directly alters tumor-associated biomineralization is currently limited. Accordingly, extrapolation from vascular calcification to cancer should be interpreted cautiously.

This review therefore distinguishes between established evidence, emerging observations, indirect mechanistic evidence, and hypothesis-generating interpretations. We first examine the cellular and molecular components of the proposed IBI and summarize established mechanisms of nutritional regulation derived predominantly from vascular calcification and related disorders. We then consider magnesium, vitamin K_2_, polyphenols, omega-3 fatty acids, and EV-associated mechanisms in relation to pathological biomineralization. Finally, we evaluate whether the biological similarities between vascular calcification and tumor-associated biomineralization provide a rational basis for extending the IBI framework to cancer and identify the experimental evidence required to test this hypothesis.

**Scope and limitations of the narrative review**

This article is a narrative review designed to integrate mechanistic evidence across nutritional science, vascular biology, immunology, extracellular vesicle research, biomineralization, and oncology. Literature was selected to provide representative experimental, mechanistic, observational, and clinical evidence relevant to the proposed IBI framework rather than according to a formal systematic-review protocol. Consequently, the literature-selection process is subject to potential selection and publication bias, and the level of evidence varies substantially among nutritional factors and disease contexts. Particular caution is therefore required when extrapolating findings from vascular calcification to tumor-associated biomineralization. The proposed IBI should consequently be regarded as a conceptual and hypothesis-generating framework requiring prospective experimental validation, rather than as an established mechanistic model.

## 2. The Proposed Immuno–Biomineral Interface

The Immuno–Biomineral Interface (IBI) is proposed here as a conceptual framework for describing the interactions among immune cells, extracellular vesicles (EVs), extracellular matrix (ECM), mineral-associated proteins, and developing mineral deposits during pathological biomineralization. The framework is based primarily on evidence from vascular calcification and related mineralizing disorders, in which inflammatory signaling, cellular phenotypic changes, ECM remodeling, and calcium–phosphate metabolism have been shown to interact during mineral deposition [17,18]. Whether an equivalent interface operates in tumor-associated biomineralization remains uncertain and requires direct experimental investigation.

The proposed IBI should therefore not be interpreted as a discrete anatomical structure or a single molecular pathway. Rather, it represents a dynamic biological interface in which cellular, extracellular, metabolic, and physicochemical processes converge. This distinction is important because the individual components of the framework are supported by different levels of evidence. Interactions among immune cells, EVs, ECM, and mineral formation are relatively well characterized in vascular calcification, whereas their coordinated regulation by nutrition and their relevance to tumor-associated biomineralization remain less established.

### 2.1. Cellular and Extracellular Components

The proposed IBI comprises several interacting cellular and extracellular components, including macrophages and other immune cells, vascular or stromal cells, EVs, apoptotic bodies, ECM proteins, mineral-associated proteins, calciprotein particles, and calcium–phosphate species. Their relative contribution varies according to tissue type and disease state [18,19].

Macrophages are particularly relevant because they respond dynamically to inflammatory mediators, metabolic signals, ECM composition, and tissue injury. Depending on the local microenvironment, macrophages can adopt functionally distinct states that influence cytokine production, oxidative stress, efferocytosis, matrix remodeling, and tissue repair. Experimental studies in vascular disease and other mineralizing environments indicate that macrophage activity can influence pathways associated with pathological calcification, although the relationship between macrophage phenotype and mineral deposition is highly context dependent [19,20].

EVs constitute another important component of the proposed interface. In vascular calcification, extracellular vesicles released by vascular cells can concentrate calcium and phosphate and provide membrane-associated sites that facilitate mineral nucleation. EVs also transport proteins, lipids, nucleic acids, and signaling molecules between cells, thereby influencing inflammatory and matrix-remodeling responses [2,15]. These dual signaling and mineral-associated functions make EVs biologically relevant to the IBI.

However, it is important to distinguish established EV participation in pathological mineralization from the more speculative proposition that dietary factors regulate mineralization primarily through EV-mediated mechanisms. Although nutritional status can modify EV abundance and cargo, direct evidence linking a defined nutritional intervention to EV-mediated mineral nucleation remains limited.

The ECM provides an additional structural component of the proposed interface. Collagen, phosphoproteins, proteoglycans, and other matrix-associated molecules influence cellular adhesion, tissue mechanics, calcium binding, and mineral nucleation. Changes in matrix composition can therefore alter the physicochemical environment in which mineral deposition occurs [18,21,22]. These observations support the concept of a cell–matrix–mineral continuum but do not imply that identical mechanisms operate across all tissues.

### 2.2. Dynamic Cell–Matrix–Mineral Signaling

Interactions within the proposed Immuno–Biomineral Interface occur through coordinated communication between cells, extracellular matrix (ECM), and mineral-associated structures. Immune cells respond to changes in extracellular architecture, matrix composition, and mineral organization through adhesion receptors, signaling complexes, and cytoskeletal pathways that regulate cellular activation and mechanosensing. These interactions allow cells to integrate biochemical and physical information from their surrounding microenvironment [17,23,24].

The mechanical properties of mineralized tissues represent an additional layer of regulation. Matrix stiffness, surface topography, crystal morphology, and mineral composition can influence cell adhesion, receptor signaling, and cellular activation. Thus, immune-cell behavior is shaped not only by soluble inflammatory mediators but also by the biophysical characteristics of the surrounding ECM and mineralized environment [25].

Protein–mineral interactions provide another important component of this interface. Matrix-associated proteins, including collagen, phosphoproteins, and mineral-binding peptides, can influence calcium–phosphate nucleation, crystal organization, and mineral maturation while also contributing to cellular adhesion and signaling [18,21,22]. These interactions illustrate how ECM composition and mineral organization may jointly influence cellular behavior and mineral formation.

Together, these observations support a cell–matrix–mineral continuum in which biochemical and biophysical signals interact during tissue remodeling and mineral formation. However, their integration into a unified Immuno–Biomineral Interface remains a conceptual interpretation rather than an experimentally established model. Whether similar cell–matrix–mineral interactions operate in tumor-associated biomineralization remains uncertain and requires direct experimental investigation.

### 2.3. Immune–Mineral Feedback

Inflammation and pathological mineralization can interact through reciprocal mechanisms. In vascular calcification, inflammatory mediators, oxidative stress, cellular injury, and matrix remodeling can promote phenotypic changes that favor mineral deposition. Conversely, mineralized microenvironments may alter cellular adhesion, mechanosensing, inflammatory signaling, and tissue architecture. These interactions provide a biological basis for considering immune and mineral processes as components of a common regulatory environment [15].

Evidence from vascular calcification, biomaterial integration, regenerative medicine, and developmental biology suggests that immune activation can precede or accompany organized mineral deposition. During physiological biomineralization, immune-like haemocytes cooperate with specialized epithelial cells to regulate calcium transport, extracellular matrix assembly, and crystal formation during shell development. Such observations provide an example of the broader involvement of immune-related cellular processes in mineralized tissue formation, although their relevance to pathological mammalian calcification remains uncertain [26].

Importantly, however, the direction and magnitude of these interactions are contextdependent. Mineral deposition should not be assumed to be uniformly pro-inflammatory, nor should inflammatory activation necessarily be interpreted as a direct cause of mineralization. Rather, the relationship may involve feedback between tissue injury, cellular phenotype, matrix remodeling, and mineral accumulation.

Collectively, these observations provide a conceptual basis for considering pathological biomineralization within a proposed Immuno–Biomineral Interface characterized by interactions among immune cells, extracellular vesicles, extracellular matrix components, and mineralizing structures. However, the extent to which these interactions constitute a unified regulatory interface remains to be experimentally established, particularly in tumor-associated biomineralization [17].

### 2.4. What Is Established Versus Hypothetical

The biological processes incorporated into the proposed Immuno–Biomineral Interface—including immune activation, extracellular matrix remodeling, extracellular vesicle-mediated communication, and calcium–phosphate regulation—are supported by substantial evidence, particularly from studies of vascular calcification [15,17,18,19]. However, the IBI itself is a conceptual and hypothesis-generating framework rather than an experimentally established biological entity.

Evidence that nutritional factors influence these individual processes is also established to varying degrees [10,11,12,13], but whether these effects converge to regulate a unified IBI remains uncertain. Most importantly, direct evidence that nutritional interventions alter tumor-associated biomineralization is currently limited. Accordingly, vascular calcification provides the primary mechanistic foundation of the framework, whereas its extension to tumor-associated biomineralization represents a testable conceptual hypothesis.

## 3. Nutritional Mechanisms Regulating Biomineralization

Nutrition may act as an important systemic modifier of biomineralization because it influences mineral availability, endocrine signaling, cellular metabolism, and immune homeostasis. Rather than acting through a single pathway, dietary factors can influence calcium and phosphate balance, oxidative stress, inflammatory responses, extracellular matrix remodeling, and extracellular vesicle biology [10,11,12]. The major nutritional regulators and their principal mechanisms within the proposed Immuno–Biomineral Interface are summarized in Table 1.

### 3.1. Regulation of Calcium–Phosphate Homeostasis

Maintenance of extracellular calcium and phosphate concentrations depends on coordinated regulation by the intestine, skeleton, kidneys, and endocrine system, principally through parathyroid hormone (PTH), fibroblast growth factor 23 (FGF23), and vitamin D [11,12,13]. These integrated regulatory networks preserve mineral homeostasis while supporting skeletal remodeling and numerous calcium-dependent cellular processes.

Dietary intake directly influences this equilibrium. Excess phosphate consumption, altered calcium intake, impaired renal phosphate clearance, or disturbances in vitamin D metabolism increase extracellular calcium–phosphate supersaturation and favor pathological mineral deposition. Hyperphosphatemia is particularly important because elevated phosphate stimulates osteogenic differentiation, extracellular matrix remodeling, and calcium–phosphate nucleation in multiple tissues [27,28,29,30].

### 3.2. Nutritional Control of Pathological Biomineralization

Pathological biomineralization is increasingly recognized as a biologically regulated process rather than passive physicochemical precipitation. Nutritional disturbances may influence the propensity for mineral deposition by altering extracellular mineral saturation, endocrine regulation, matrix composition, and endogenous inhibitors of calcification, including fetuin-A, matrix Gla protein, and osteopontin [2,14].

Experimental and clinical studies demonstrate that chronic disruption of calcium–phosphate metabolism promotes ectopic mineralization in blood vessels, kidneys, heart valves, and other soft tissues. These pathological processes arise through coordinated interactions among inflammatory signaling, extracellular matrix remodeling, collagen organization, extracellular vesicles, and osteogenic differentiation, collectively generating permissive environments for mineral nucleation and crystal maturation [3,5,7,15,16].

Although direct evidence linking nutritional imbalance to tumor-associated biomineralization remains limited, these established mechanisms provide a biological rationale for investigating whether similar nutritional influences operate within tumor microenvironments.

### 3.3. Intracellular Calcium Overload and Calcification Signaling

Intracellular calcium homeostasis represents an additional cellular layer through which mineral stress may influence pathological calcification. Sustained disturbances in extracellular calcium and phosphate can alter cellular calcium handling and promote intracellular Ca^2+^ accumulation, with downstream effects on mitochondrial function, oxidative stress, and mineralization-related cellular responses. In osteogenic cells, phosphate-induced mineralization has been associated with increased reactive oxygen species (ROS) generation, supporting a link between mineral stress, oxidative signaling, and acquisition of a mineralization-permissive phenotype. Persistent calcium and phosphate stress may therefore integrate metabolic and oxidative signals with inflammatory and osteogenic pathways, while extracellular vesicle release may provide an additional mechanism through which stressed cells contribute to extracellular mineral deposition [1,31,32].

In vascular cells, altered intracellular Ca^2+^ signaling can influence cellular phenotype through spatially organized calcium-dependent signaling networks. Ca^2+^ microdomains formed around ion channels, intracellular calcium stores, and downstream signaling proteins can couple changes in cytosolic Ca^2+^ to transcriptional and longer-term cellular responses. In vascular smooth muscle cells, Ca^2+^-dependent activation of Ca^2+^/calmodulin-dependent kinases (CaMKs) and calcineurin can regulate gene expression, including transcription of transient receptor potential canonical (TRPC) channels, thereby contributing to feedback regulation of cellular Ca^2+^ homeostasis [33,34]. Contemporary evidence further indicates that Ca^2+^ microdomain-based excitation–transcription coupling involving CaMK and calcineurin can participate in vascular smooth muscle remodeling and pathological cardiovascular responses [34]. These mechanisms provide a plausible signaling framework through which persistent calcium dysregulation could influence cellular differentiation, extracellular matrix remodeling, and other processes relevant to pathological mineralization.

Importantly, these calcium-dependent signaling mechanisms should not be interpreted as direct evidence that intracellular calcium overload itself drives pathological calcification in every biological context. Rather, they represent a potential mechanistic bridge linking extracellular mineral stress to intracellular signaling, oxidative responses, cellular phenotype, and extracellular matrix changes. The extent to which nutritional factors directly regulate intracellular Ca^2+^ overload or CaMK/calcineurin signaling during pathological biomineralization remains incompletely defined. This distinction is particularly important when extrapolating mechanisms established in vascular calcification to tumor-associated biomineralization, where calcium signaling may interact with oxidative, inflammatory, metabolic, and extracellular-vesicle pathways within the tumor microenvironment.

### 3.4. Oxidative Stress and Redox Regulation

Reactive oxygen species (ROS) function as essential signaling molecules during physiological mineral metabolism, regulating osteoblast differentiation, extracellular matrix maturation, and bone remodeling. Persistent oxidative stress, however, disrupts these homeostatic processes by promoting inflammatory activation, extracellular matrix remodeling, osteogenic differentiation, and pathological calcium–phosphate deposition [35,36].

Elevated extracellular phosphate enhances mitochondrial ROS production and activates signaling pathways controlling osteogenic transcription, kinase activation, and cellular metabolism, thereby promoting pathological mineralization [31,37]. Oxidative stress also stimulates matrix-degrading enzymes and inflammatory cytokines that remodel the extracellular environment to facilitate mineral nucleation and crystal growth [38,39].

Diet-derived antioxidants and bioactive phytochemicals counteract these effects by restoring redox homeostasis and suppressing oxidative stress-dependent inflammatory signaling. The magnitude and clinical relevance of these effects, however, vary among individual compounds and disease contexts [40].

### 3.5. Immunometabolic Regulation of Biomineralization

Nutrition also regulates biomineralization through coordinated effects on immune function and tissue metabolism. Dietary bioactive compounds influence macrophage polarization, cytokine production, inflammasome activation, and cellular metabolic programming, thereby shaping inflammatory environments that determine mineralization propensity [41,42,43,44,45].

Macrophages represent an important cellular component of this nutritional–inflammatory axis. Polyphenols, omega-3 polyunsaturated fatty acids, and numerous plant-derived metabolites promote anti-inflammatory macrophage polarization while suppressing NF-κB-, STAT-, and Toll-like receptor-dependent inflammatory pathways [43,44,45]. These nutritional interventions reduce production of TNF-α, IL-1β, and IL-6 while enhancing anti-inflammatory mediators, thereby modifying the inflammatory microenvironment in which biomineralization develops.

Several dietary compounds also suppress activation of the NLRP3 inflammasome through improved mitochondrial function and enhanced autophagy, providing an additional mechanistic link between nutrition, inflammation, and mineral metabolism [46,47].

### 3.6. Transition to Nutrient-Specific Regulation

These pathways provide the mechanistic basis for examining individual nutritional modulators within the Immuno–Biomineral Interface. The following sections examine magnesium, vitamin K_2_, polyphenols, omega-3 fatty acids, and nutrition-associated regulation of extracellular vesicles, with emphasis on the strength of evidence supporting their roles in pathological biomineralization and the extent to which these mechanisms may be relevant to tumor-associated biomineralization.

## 4. Magnesium as a Nutritional Modifier of Pathological Biomineralization

Magnesium is one of the most extensively studied endogenous inhibitors of pathological biomineralization. Experimental, epidemiological, and clinical evidence indicates that adequate magnesium availability can limit ectopic calcium–phosphate deposition, particularly in the cardiovascular system and chronic kidney disease (CKD), whereas magnesium deficiency is associated with increased vascular calcification, endothelial dysfunction, arterial stiffness, and adverse cardiovascular outcomes [48,49,50,51].

Unlike nutritional regulators that act primarily through endocrine pathways, magnesium exerts complementary physicochemical and biological effects across the Immuno–Biomineral Interface. It can directly interfere with calcium–phosphate crystal formation while also modulating inflammatory signaling, oxidative stress, extracellular matrix remodeling, and osteogenic cell differentiation. These complementary actions make magnesium an important nutritional modifier of pathways associated with pathological mineralization [49].

### 4.1. Inhibition of Mineral Nucleation

A major anti-calcific action of magnesium is its ability to interfere with calcium–phosphate crystal nucleation and hydroxyapatite maturation. By stabilizing less crystalline mineral phases and limiting crystal growth, magnesium can restrict the progression of ectopic calcification before mature mineral deposits become established [32,52].

Experimental studies further demonstrate that magnesium suppresses phosphate-induced mineral precipitation independently of intracellular magnesium transport through TRPM7 channels, indicating that this protective effect largely reflects direct extracellular regulation of mineral chemistry rather than cell-mediated signaling [52].

### 4.2. Regulation of Cellular Phenotype

Magnesium also influences the cellular reprogramming that precedes pathological biomineralization. Elevated phosphate concentrations induce osteogenic transdifferentiation of vascular smooth muscle cells (VSMCs), characterized by increased expression of RUNX2, osteopontin, alkaline phosphatase, and other bone-associated proteins. Magnesium suppresses this phenotypic conversion while preserving the contractile VSMC phenotype, thereby limiting extracellular matrix mineralization [53].

These findings indicate that magnesium regulates not only mineral crystal formation but also the biological programs responsible for creating mineralization-permissive microenvironments.

### 4.3. Magnesium and the Immuno–Biomineral Interface

Beyond its direct effects on mineral formation, magnesium influences several components of the Immuno–Biomineral Interface. Magnesium deficiency promotes oxidative stress, endothelial dysfunction, inflammatory cytokine production, and extracellular matrix remodeling, processes that can favor pathological calcification [54,55].

Conversely, adequate magnesium availability can attenuate reactive oxygen species generation, preserve endothelial integrity, and suppress inflammatory activation. These effects are particularly relevant in CKD, where chronic inflammation, hyperphosphatemia, and oxidative stress interact to promote vascular calcification. Experimental and clinical studies suggest that magnesium can partially counteract phosphate toxicity and may reduce calcification progression through simultaneous effects on mineral chemistry and inflammatory pathways [48,49,56].

### 4.4. Translational Perspectives on Magnesium

Observational studies consistently report an inverse association between circulating magnesium concentrations and vascular calcification, cardiovascular events, and mortality, particularly in patients with CKD [48,51]. Experimental models further support the protective effects of magnesium supplementation, and ongoing clinical studies are evaluating whether correction of magnesium deficiency can reduce arterial stiffness and calcific disease progression in high-risk populations [57].

Although the available evidence derives predominantly from cardiovascular and renal disease, the underlying mechanisms provide a biologically plausible framework for considering magnesium within broader pathological biomineralization processes. Direct evidence for an effect on tumor-associated biomineralization is currently lacking.

## 5. Vitamin K_2_ as a Regulator of Mineral Homeostasis and the Immuno–Biomineral Interface

Vitamin K_2_ (menaquinone) is an important nutritional factor involved in calcium homeostasis and the regulation of pathological biomineralization through activation of vitamin K-dependent proteins involved in extracellular calcium handling. Unlike calcium and phosphate, which provide substrates for mineral formation, vitamin K_2_ promotes the γ-carboxylation of endogenous calcification inhibitors while supporting physiological skeletal mineralization [58].

Beyond its established role in vascular calcification and bone metabolism, emerging evidence suggests that vitamin K-dependent pathways may influence components of the proposed Immuno–Biomineral Interface, including mineral nucleation, extracellular matrix organization, and inflammatory signaling [59,60]. However, most evidence for these effects is derived from vascular and skeletal systems, and their relevance to tumor-associated biomineralization remains indirect.

### 5.1. Activation of Endogenous Calcification Inhibitors

The principal anti-calcific action of vitamin K is the activation of matrix Gla protein (MGP), an important endogenous inhibitor of ectopic mineralization. Following γ-carboxylation, MGP interacts with calcium and nascent hydroxyapatite crystals and contributes to the inhibition of crystal growth and calcium–phosphate deposition within soft tissues. In contrast, vitamin K deficiency increases the proportion of inactive under-carboxylated MGP and has been associated with accelerated vascular calcification and cardiovascular disease [58,61].

Vitamin K_2_ also promotes the γ-carboxylation of osteocalcin, a protein involved in physiological bone mineralization. Through the coordinated activation of MGP and osteocalcin, vitamin K-dependent pathways contribute to the regulation of mineral distribution between skeletal and extraskeletal tissues [62,63].

### 5.2. Regulation of Biomineralization

Experimental and clinical evidence indicates that vitamin K-dependent pathways can influence pathological biomineralization through several complementary mechanisms. In addition to its effects on MGP activity, vitamin K-dependent pathways have been associated with regulation of vascular cell phenotype, extracellular matrix mineralization, and tissue remodeling [59,64].

Clinical studies have associated inadequate vitamin K status with increased vascular calcification, particularly in populations with chronic kidney disease and cardiovascular disease. This relationship has also been discussed in the context of the “calcium paradox,” in which vascular calcium accumulation may occur alongside impaired skeletal mineralization [61,65]. These findings support a role for vitamin K in mineral homeostasis, although the extent to which vitamin K_2_ supplementation can prevent or reverse vascular calcification remains an area of ongoing investigation [62].

### 5.3. Vitamin K_2_ and the Immuno–Biomineral Interface

Vitamin K-dependent pathways may also influence extracellular matrix remodeling and vascular smooth muscle cell phenotype, processes that are relevant to pathological calcification [59,60].

Vitamin K-dependent regulation of matrix proteins may contribute to maintenance of extracellular matrix organization and a tissue environment that is less permissive to calcium–phosphate deposition. These observations provide a potential mechanistic link between vitamin K status and the proposed Immuno–Biomineral Interface, although direct evidence for an integrated effect on immune, matrix, and mineralization pathways remains limited.

### 5.4. Translational Perspectives on Vitamin K_2_

Most evidence supporting the potential protective effects of vitamin K_2_ derives from studies of vascular calcification, chronic kidney disease, and bone biology. Whether similar mechanisms regulate biomineralization within inflammatory lesions or tumor-associated microenvironments remains largely unknown. Thus, the potential relevance of vitamin K_2_ to tumor-associated biomineralization should currently be considered indirect and hypothesis-generating rather than established. The proposed connection is based on the involvement of vitamin K-dependent pathways in mineral regulation and tissue remodeling rather than on direct demonstrations of nutritional modulation of tumor mineralization [61,62].

Evidence strength: Evidence for vitamin K-dependent regulation of mineral homeostasis and vascular calcification is supported by experimental and clinical studies, whereas evidence linking vitamin K_2_ directly to tumor-associated biomineralization remains limited and hypothesis-generating.

## 6. Polyphenols and Flavonoids as Modulators of the Immuno–Biomineral Interface

Plant-derived polyphenols and flavonoids have emerged as potential nutritional modulators of pathological biomineralization because they simultaneously influence oxidative stress, inflammatory signaling, cellular differentiation, and extracellular matrix remodeling. Although much of the mechanistic evidence derives from vascular calcification, bone remodeling, and chronic inflammatory diseases, these compounds target biological pathways involved in ectopic calcium–phosphate deposition. Rather than directly dissolving established mineral deposits, polyphenols may modify the tissue microenvironment preceding mineral nucleation by attenuating oxidative injury, suppressing inflammatory activation, and limiting osteogenic phenotypic switching [6,7,66].

### 6.1. Regulation of Oxidative Stress and Inflammatory Signaling

Oxidative stress is an important contributor to pathological biomineralization, promoting osteogenic differentiation, extracellular matrix remodeling, and chronic inflammation. Plant-derived polyphenols—including curcumin, epigallocatechin gallate (EGCG), quercetin, and resveratrol—can counteract these processes through coordinated regulation of antioxidant and inflammatory signaling pathways.

These compounds can activate the Nrf2 antioxidant pathway, increasing expression of cytoprotective enzymes including heme oxygenase-1 (HO-1), NAD(P)H quinone oxidoreductase-1 (NQO1), superoxide dismutase (SOD), and catalase, thereby limiting reactive oxygen species accumulation. Concurrently, they can suppress NF-κB-dependent inflammatory signaling and reduce production of pro-inflammatory cytokines including TNF-α, IL-1β, and IL-6 [66,67]. Curcumin additionally inhibits NF-κB, PI3K–AKT, and ERK signaling in models of calcification involving human aortic valve interstitial cells [68] whereas resveratrol has been reported to activate the SIRT1–Nrf2 axis, preserve mitochondrial function and cellular redox homeostasis, and attenuate vascular calcification. Collectively, these actions may generate a tissue microenvironment that is less permissive to pathological mineral nucleation.

### 6.2. Regulation of Cellular Reprogramming

Polyphenols may further influence biomineralization by limiting osteogenic transdifferentiation, a characteristic feature of ectopic calcification. Curcumin suppresses expression of RUNX2, alkaline phosphatase (ALP), and other osteogenic regulators while modulating PI3K–AKT and ERK signaling pathways implicated in vascular calcification [68]. Experimental studies further indicate that controlled-release curcumin can attenuate ectopic calcification by influencing progenitor-cell differentiation and reducing mineral deposition [69].

Resveratrol similarly attenuates phosphate-induced osteogenic differentiation by downregulating RUNX2, BMP2, and ALP through modulation of Wnt/β-catenin, AMPK, and JNK signaling pathways, thereby reducing calcium deposition in vascular smooth muscle cells [70,71,72]. Although direct evidence for EGCG and quercetin in pathological biomineralization is comparatively limited, both compounds modulate oxidative stress- and inflammation-dependent pathways involved in osteogenic differentiation, providing a potential mechanistic basis for broader anti-calcific effects.

### 6.3. Translational Perspectives on Polyphenols

Beyond their antioxidant activities, polyphenols can modulate components of the Immuno–Biomineral Interface by influencing innate immune responses and inflammatory signaling. Curcumin has been reported to promote macrophage polarization toward reparative M2 phenotypes while suppressing pro-inflammatory activation, potentially creating an osteo-immunological environment less conducive to inflammatory remodeling [73]. Quercetin similarly suppresses inflammatory signaling through inhibition of NF-κB-dependent pathways [74].

Resveratrol attenuates activation of the NLRP3 inflammasome, reducing pyroptotic signaling and IL-1β production in vascular smooth muscle cells while limiting calcification [75]. EGCG has also been reported to suppress NF-κB- and NLRP3-dependent inflammatory signaling, suggesting additional mechanisms through which green tea catechins may influence mineralization-permissive microenvironments [76].

Because macrophages contribute to extracellular matrix remodeling, cytokine production, and osteogenic signaling, these immunomodulatory effects may indirectly influence the formation of mineralization-permissive niches and thereby affect ectopic calcium–phosphate deposition [77,78].

### 6.4. Polyphenols: Translational Perspectives

Current evidence for the anti-calcific effects of polyphenols remains predominantly experimental, with relatively limited clinical evidence directly evaluating nutritional modulation of pathological biomineralization. Evidence linking individual polyphenols to tumor-associated biomineralization is also lacking or indirect. Accordingly, their potential relevance to the Immuno–Biomineral Interface in tumors should be considered hypothesis-generating rather than established.

## 7. Omega-3 Fatty Acids as Immunometabolic Regulators of Biomineralization

Omega-3 polyunsaturated fatty acids (PUFAs) are important nutritional modulators of inflammation and immune homeostasis that may indirectly influence pathological biomineralization. Unlike magnesium or vitamin K_2_, omega-3 fatty acids are not established as direct inhibitors of calcium–phosphate crystal formation. Instead, they influence the inflammatory microenvironment through coordinated modulation of macrophage polarization, cytokine production, inflammation resolution, and immunometabolic signaling, thereby potentially modifying the biological conditions that influence mineral deposition [79,80,81].

### 7.1. Resolution of Inflammation

One of the best-characterized actions of omega-3 fatty acids is the promotion of macrophage polarization toward anti-inflammatory M2 phenotypes while suppressing pro-inflammatory M1 activation. Experimental studies demonstrate that these effects involve activation of multiple signaling pathways, including AMPK/SIRT1, JAK2/STAT3, and PPAR-γ, which collectively suppress inflammatory activation, improve cellular metabolic homeostasis, and promote tissue repair [82,83,84,85].

Omega-3 fatty acids also serve as precursors of specialized pro-resolving mediators (SPMs), including resolvins, protectins, and maresins. Unlike conventional anti-inflammatory mechanisms that primarily suppress immune activation, SPMs actively orchestrate the resolution phase of inflammation by enhancing efferocytosis, limiting neutrophil recruitment, and restoring tissue homeostasis. These actions may reduce the chronic inflammatory microenvironments that facilitate pathological biomineralization [86,87,88,89].

### 7.2. Cytokine Networks and Tissue Microenvironments

Omega-3 fatty acids can suppress production of pro-inflammatory cytokines, including TNF-α, IL-1β, and IL-6, while increasing anti-inflammatory mediators such as IL-10, through modulation of NF-κB and Toll-like receptor (TLR) signaling pathways [79,82,90]. These immunomodulatory effects may attenuate oxidative stress and chronic inflammatory activation associated with extracellular matrix remodeling and osteogenic differentiation, thereby generating tissue environments that are potentially less permissive to ectopic calcium–phosphate deposition [6,67,80].

### 7.3. Integration Within the Immuno–Biomineral Interface

Current evidence suggests that omega-3 fatty acids can influence multiple components of the proposed Immuno–Biomineral Interface. Through modulation of macrophage phenotype, inflammatory resolution, cytokine signaling, and cellular metabolism, they may indirectly influence extracellular matrix remodeling, osteogenic signaling, and conditions favoring mineral nucleation [80,81,84]. Their capacity to generate specialized pro-resolving mediators distinguishes omega-3 fatty acids from nutritional factors that primarily suppress inflammatory signaling, as SPMs actively promote restoration of tissue homeostasis [87].

Direct evidence linking omega-3 fatty acids to extracellular vesicle (EV)-mediated biomineralization remains limited. Nevertheless, because macrophage phenotype and inflammatory signaling can influence EV biogenesis, cargo composition, and intercellular communication, nutritional regulation of EV biology represents a plausible mechanism connecting omega-3 fatty acids with pathological biomineralization [91,92].

### 7.4. Translational Perspectives

Most mechanistic evidence relevant to the potential anti-calcific effects of omega-3 fatty acids derives from studies of vascular disease, atherosclerosis, and chronic inflammatory conditions [80]. Direct evidence linking omega-3 fatty acids to tumor-associated biomineralization is currently lacking. Their potential relevance to the Immuno–Biomineral Interface in tumors therefore remains mechanistically plausible but hypothesis-generating, particularly through effects on inflammatory signaling, macrophage responses, and oxidative stress (Table 2).

## 8. Extracellular Vesicles as a Potential Nutrient-Sensitive Link

### 8.1. Nutritional Regulation of EV Biogenesis and Release

Dietary composition can influence circulating extracellular vesicle (EV) populations, including their abundance and cellular origin. Nutritional factors affect metabolic and inflammatory states that are associated with changes in EV release. For example, diets enriched in omega-3 polyunsaturated fatty acids have been reported to modify circulating EV profiles and inflammatory phenotypes, whereas excessive sodium intake has been associated with increased endothelial-derived EVs carrying adhesion and inflammatory molecules [93,94]. These findings support an association between nutritional status and EV biology; however, whether such changes directly alter pathological mineralization remains uncertain. Thus, nutrition–EV interactions should be considered an emerging regulatory component of the Immuno–Biomineral Interface rather than an established causal pathway linking diet to biomineralization [79,95,96,97].

### 8.2. Diet-Dependent Remodeling of EV Cargo

Nutritional factors may also influence the molecular cargo of EVs, including proteins, lipids, metabolites, and regulatory RNAs. Experimental studies indicate that dietary lipids, vitamins, and food-derived bioactive compounds can modify EV-associated molecular profiles and thereby influence pathways involved in inflammation, cellular stress, and extracellular matrix remodeling. Plant- and food-derived vesicles have additionally been investigated as carriers of bioactive molecules capable of modulating host cellular responses and epithelial homeostasis [95,98,99,100,101]. These observations establish that nutritional signals can influence vesicle-mediated intercellular communication. However, evidence that diet-dependent changes in EV cargo directly regulate pathological mineral deposition remains limited. The relevance of such cargo remodeling to tumor-associated biomineralization is therefore currently hypothesis-generating rather than established.

### 8.3. EVs as Mineralization Organelles

A stronger body of evidence supports a role for specialized extracellular vesicles in mineral formation. In physiologic mineralizing tissues, matrix vesicles provide confined microenvironments that facilitate calcium–phosphate accumulation and hydroxyapatite nucleation. Their mineralizing activity is regulated by membrane-associated transporters, phosphatases, and other proteins involved in phosphate metabolism and crystal formation. Comparable vesicle-mediated processes have been described in pathological calcification, including vascular calcification, where extracellular vesicles released by vascular smooth muscle cells can contribute to ectopic mineral nucleation [91,102,103,104,105].

These findings establish EV-mediated mineralization as a biologically plausible mechanism in several mineralizing and calcifying contexts. Nevertheless, evidence from vascular and skeletal systems should not be assumed to apply directly to tumor-associated biomineralization. Whether tumor-derived or stromal EVs undergo comparable mineralizing functions, and whether these processes are nutritionally modifiable, remain incompletely defined.

### 8.4. Immune–EV Interactions Within the Immuno–Biomineral Interface

EVs also participate in intercellular immune communication. Immune-cell-derived EVs can influence macrophage phenotypes, cytokine signaling, endothelial responses, and extracellular matrix remodeling, while nutritional interventions have been reported to modify some aspects of EV-mediated inflammatory signaling. Food-derived and plant-derived vesicles have likewise been investigated for their capacity to influence epithelial and inflammatory pathways [92,99,100,101,106]. These observations provide separate lines of evidence linking nutrition, EV biology, and immune regulation.

Whether these processes converge to regulate mineral deposition in vivo remains unresolved. In particular, the proposed sequence in which dietary factors alter immune-cell or stromal EV production or cargo, followed by changes in EV-mediated mineral nucleation within a tumor microenvironment, has not been directly established. EVs should therefore be regarded as a potential mechanistic bridge within the Immuno–Biomineral Interface rather than as a demonstrated mediator of nutrition-dependent tumor biomineralization.

### 8.5. Evidence Boundaries and Future Perspectives

Current evidence supports several components of the proposed framework, but at different levels of certainty. Nutritional status can influence EV abundance and molecular composition, and EVs have established roles in intercellular communication and, in specific tissues, mineral nucleation. In contrast, direct evidence that dietary interventions alter EV-mediated pathological mineralization is limited, and evidence specifically connecting nutritional modulation of EVs with tumor-associated biomineralization remains particularly sparse.

Accordingly, the nutrition–EV–biomineralization axis should be considered a hypothesis-generating component of the Immuno–Biomineral Interface. Future studies should first establish whether defined nutritional exposures reproducibly alter the abundance, cellular origin, cargo, and mineral-associated properties of EVs in calcifying tissues. In tumor models, these studies could be complemented by spatially resolved transcriptomic and proteomic approaches, metabolomic profiling, EV characterization, and high-resolution analysis of mineral composition and localization. Such approaches would help determine whether the mechanisms established in vascular calcification extend to tumor-associated biomineralization, rather than assuming mechanistic equivalence between these settings [97,99].

## 9. Direct Evidence and Nutritional Context of Tumor-Associated Biomineralization

### 9.1. Tumor-Associated Mineralization as a Heterogeneous Pathological Phenomenon

Tumor-associated mineralization is documented in several malignancies, particularly breast and thyroid cancers, but its composition, cellular origin, and biological significance vary considerably among tumor types and pathological contexts [8,9]. Breast microcalcifications provide one of the best-characterized examples. Early studies suggested that mineral deposits associated with breast cancer may involve active mineralization by malignant cells rather than representing exclusively passive or degenerative calcification [107,108]. Nevertheless, tumor-associated calcification is heterogeneous, and the presence of mineral deposits alone does not demonstrate a regulated biomineralization programme.

Chemical characterization has further demonstrated that breast microcalcifications are compositionally diverse. Raman spectroscopic analysis identified differences in the mineral composition of calcifications associated with benign and malignant breast lesions, supporting the possibility that mineral characteristics reflect differences in the surrounding tissue microenvironment [109]. Subsequent studies have similarly highlighted relationships among mineral composition, tissue pathology, and biochemical features during breast cancer progression [110]. These findings indicate that tumor-associated mineralization should not be considered a single uniform process and emphasize the importance of characterizing mineral type, morphology, localization, and associated tissue features when investigating its biological significance.

### 9.2. Evidence for Regulated Cellular and Molecular Components

Several experimental observations support the possibility that at least a subset of tumor-associated mineral deposits may arise through regulated cellular processes. Comparisons between breast microcalcification and physiological mineralization have identified potential roles for mineral-regulatory pathways, extracellular matrix interactions, cellular phenotype changes, and local calcium–phosphate conditions [111]. In experimental models, tumorigenic mammary cells have demonstrated the capacity to generate mineral deposits, providing evidence that mammary mineralization can involve active cellular mechanisms [112].

Mechanistic studies have further implicated alkaline phosphatase (ALP) activity, phosphate handling, osteogenic-associated gene expression, and calcium-related signaling in this process. Cox et al. demonstrated molecular features consistent with regulated mammary mineralization and investigated potential functional consequences of hydroxyapatite deposition in breast cancer [112]. O’Grady and Morgan subsequently showed that calcium deposition in an in vitro model of human breast tumor calcification was associated with ALP activity, altered expression of osteogenic genes, and dysregulation of the TRPM7 ion channel [113]. Histopathological evidence has also identified epithelial cells with mesenchymal and osteogenic-associated characteristics in relation to breast microcalcifications [114].

Together, these studies strengthen the concept that some forms of tumor-associated mineralization may contain actively regulated cellular components. However, they do not establish that all tumor calcifications arise through the same mechanism. Dystrophic mineralization, cellular injury, necrosis, altered extracellular physicochemistry, and other processes may contribute in different proportions depending on tumor type and stage. Furthermore, mechanisms identified in breast cancer should not automatically be generalized to other malignancies.

### 9.3. Relationship to the Immuno–Biomineral Interface

The direct tumor literature provides several potential points of intersection with the proposed Immuno–Biomineral Interface. Tumor-associated mineralization occurs within a complex microenvironment containing malignant cells, stromal cells, immune populations, extracellular matrix components, extracellular vesicles, and altered metabolic and ionic conditions. The evidence for cellular mineralization, osteogenic-associated phenotypes, ALP activity, phosphate handling, and calcium-related signaling in breast cancer [107,111,112,113,114] provides a biological basis for investigating whether these components interact locally during mineral formation.

Nevertheless, the strength of evidence differs substantially among individual components of the proposed IBI. In vascular calcification, extracellular vesicles can participate directly in mineral nucleation and propagation, and their mineralizing functions have been characterized experimentally [1,91,103,104]. In tumors, EV-mediated communication is well established [96,99], but direct evidence demonstrating that tumor-derived EVs initiate or regulate pathological mineral nucleation remains comparatively limited. Similarly, inflammatory and immune processes are fundamental components of the tumor microenvironment [115], but their direct contribution to the initiation or maturation of tumor mineral deposits has not been established to the same extent as in vascular calcification.

The IBI should therefore not be interpreted as evidence that vascular and tumor mineralization are mechanistically equivalent. Rather, direct observations of tumor mineralization identify specific cellular, matrix-associated, ionic, and potentially vesicular processes that can now be investigated experimentally within the IBI framework.

### 9.4. Nutritional and Metabolic Context of Tumor-Associated Biomineralization

Nutritional status may influence several biological processes relevant to the microenvironments in which pathological mineralization develops. Calcium and phosphate homeostasis, magnesium availability, vitamin K-dependent proteins, lipid metabolism, oxidative stress, inflammatory signaling, immune-cell metabolism, and EV composition can all be influenced directly or indirectly by nutritional factors [10,11,12,13,41,48,58,74,79,81,95,116]. These processes overlap with pathways implicated in vascular calcification and with several cellular and molecular features observed in tumor-associated mineralization.

However, an important evidentiary distinction is required. Evidence that magnesium, vitamin K_2_, omega-3 fatty acids, or polyphenols influence mineral metabolism, inflammation, oxidative stress, immune responses, or EV biology does not demonstrate that these nutritional factors regulate tumor-associated mineral deposition. Likewise, the identification of calcium–phosphate-related mechanisms, ALP activity, osteogenic-associated phenotypes, or altered ion-channel activity in breast tumor mineralization [111,112,113,114] does not establish nutritional control of these processes.

At present, direct experimental evidence demonstrating that defined nutritional exposures alter the initiation, quantity, composition, maturation, or spatial distribution of tumor-associated mineral deposits remains scarce. Therefore, nutritional modulation of tumor biomineralization should be considered an unresolved question rather than an established mechanism.

### 9.5. From Direct Tumor Evidence to a Testable Nutrition–IBI Hypothesis

The available literature therefore supports two related but currently incomplete evidence streams. First, vascular calcification studies provide substantial mechanistic evidence that mineral metabolism, inflammation, oxidative stress, extracellular matrix remodeling, cellular phenotype changes, and EV-mediated mineral nucleation contribute to pathological biomineralization. Several of these processes can be modified by nutritional factors, including magnesium, vitamin K_2_, omega-3 fatty acids, and polyphenols. Second, direct tumor studies demonstrate that mineral deposits are present in selected malignancies and that, particularly in breast cancer, mineralization can be associated with cellular activity, ALP, osteogenic-associated pathways, calcium and phosphate handling, and distinct mineral compositions [107,108,109,110,111,112,113,114].

The critical evidence gap lies at the intersection of these two fields. It remains unknown whether defined nutritional exposures modify tumor-associated biomineralization through systemic changes in mineral metabolism or through local effects on tumor cells, immune cells, extracellular matrix, EVs, oxidative stress, or mineral nucleation. Consequently, a causal sequence linking nutritional exposure to IBI remodeling and subsequent tumor mineralization has not been established.

This gap defines the hypothesis-generating purpose of the IBI framework. Future studies should combine controlled nutritional interventions with quantitative assessment of tumor mineral deposition and composition, spatial characterization of tumor and immune-cell phenotypes, extracellular matrix analysis, calcium and phosphate signaling, and EV profiling. Such approaches could determine whether nutritional factors modify tumor-associated biomineralization directly, indirectly through the tumor microenvironment, or not at all. Until such evidence becomes available, extrapolation from vascular calcification should remain clearly distinguished from mechanisms demonstrated directly in tumors.

## 10. From Vascular Calcification to Tumor Biomineralization: An Integrative Immuno–Biomineral Framework

Much of the mechanistic understanding of pathological biomineralization derives from vascular calcification, where disturbances in mineral metabolism, inflammation, EV biology, cellular stress, and extracellular matrix remodeling contribute to ectopic calcium–phosphate deposition [5,6,7,16,91,102,103]. As discussed in Section 9, direct studies of tumor-associated mineralization, particularly in breast cancer, additionally support roles for cellular mineralization, osteogenic-associated phenotypes, alkaline phosphatase activity, calcium and phosphate handling, and distinct mineral compositions [107,108,109,110,111,112,113,114]. Together, these findings provide a rationale for investigating points of mechanistic convergence while avoiding the assumption that vascular and tumor mineralization are equivalent processes. The IBI is therefore proposed as a hypothesis-generating framework for organizing these relationships and defining experimentally testable connections between nutrition, pathological mineralization, and tumor biology (Figure 1 and Figure 2).

### 10.1. Conserved and Context-Dependent Mechanisms of Pathological Biomineralization

Vascular tissues and tumors differ substantially in cellular composition, function, extracellular matrix organization, vascular architecture, and metabolic environment. Nevertheless, both settings can involve inflammatory signaling, oxidative and cellular stress, extracellular matrix remodeling, altered mineral handling, and EV-mediated communication. In vascular calcification, several of these processes have direct mechanistic support, including EV-associated mineral nucleation [1,91,103,104]. In tumors, evidence for regulated mineralization is emerging but remains more context-dependent, with the strongest direct evidence currently derived from breast cancer models and pathological specimens [107,108,109,110,111,112,113,114].

Within the IBI framework, these observations define candidate points of mechanistic convergence rather than conserved pathways. Their relative importance is likely to depend on tissue type, disease stage, cellular origin, extracellular matrix composition, and the physicochemical environment surrounding the developing mineral phase.

### 10.2. Nutritional Influences Across Biomineralizing Systems

Nutritional factors can modulate several processes relevant to pathological biomineralization. Magnesium influences calcium–phosphate crystallization and calcification-associated cellular responses; vitamin K-dependent pathways support endogenous inhibitors of calcification; polyphenols can modify oxidative and inflammatory signaling; and omega-3 fatty acids influence inflammatory resolution and macrophage-associated responses [48,52,58,62,68,70,74,79,80,81]. Nutritional exposures may also influence EV abundance or molecular composition in selected experimental and clinical contexts [93,94,95].

However, these observations primarily derive from vascular, renal, inflammatory, or other non-tumor systems. They should therefore be distinguished from evidence of nutritional regulation of tumor mineralization. The nutritional component of the IBI represents a potentially modifiable influence on pathways relevant to biomineralization, not a demonstrated direct regulator of tumor-associated mineral deposition.

### 10.3. Integrating Nutritional and Tumor Biomineralization Evidence

The direct tumor evidence summarized in Section 9 establishes that mineral deposition in selected malignancies can involve more than passive accumulation and may be associated with cellular and molecular processes such as ALP activity, osteogenic-associated signaling, altered ion handling, and changes in mineral composition [107,108,109,110,111,112,113,114]. Separately, nutritional studies demonstrate modulation of several pathways capable of influencing mineralizing microenvironments.

The critical question is whether these two evidence streams intersect within tumors. Specifically, can defined nutritional exposures modify immune, EV, matrix, calcium–phosphate, or mineralization-associated pathways within the tumor microenvironment, and do these changes subsequently alter the quantity, composition, maturation, or spatial distribution of tumor-associated mineral deposits? At present, this sequence has not been directly demonstrated. It therefore represents a testable prediction of the IBI framework rather than an established nutrition–tumor biomineralization pathway.

### 10.4. Evidence Boundaries and Research Priorities

The IBI framework integrates independently supported biological processes while maintaining clear boundaries between established evidence and mechanistic extrapolation. Nutritional modulation of mineral metabolism, inflammatory signaling, oxidative stress, and selected aspects of EV biology is supported by varying levels of experimental and clinical evidence. EV-associated mineral nucleation is well characterized in specific calcifying tissues, while direct tumor studies support regulated components of mineral formation in selected malignancies. What remains unvalidated is the causal pathway connecting nutritional exposure, remodeling of the tumor IBI, and subsequent changes in tumor-associated biomineralization.

Future studies should therefore test individual links within this pathway. Controlled nutritional interventions should be combined with quantitative assessment of tumor mineral burden and composition, EV abundance and cargo, immune-cell states, extracellular matrix organization, and calcium–phosphate signaling. Spatial molecular profiling and mineral characterization could determine whether nutrient-associated biological changes colocalize with developing mineral deposits. Comparative studies across vascular and tumor models would further distinguish shared mechanisms from tissue-specific processes. The major evidence gaps and experimentally testable questions are summarized in Table 3.

## 11. Conclusions and Future Perspectives

Pathological biomineralization involves interactions among mineral metabolism, cellular stress, inflammation, extracellular matrix remodeling, and extracellular vesicle (EV) biology. These processes are best characterized in vascular calcification, where substantial mechanistic evidence supports their contribution to ectopic mineral deposition. Direct studies of tumor-associated mineralization, particularly in breast cancer, additionally indicate that some mineral deposits may involve regulated cellular processes, including altered calcium and phosphate handling, alkaline phosphatase activity, osteogenic-associated phenotypes, and distinct mineral compositions [107,108,109,110,111,112,113,114]. However, the extent to which these mechanisms correspond to those established in vascular calcification remains unresolved.

Within this evidence landscape, the Immuno–Biomineral Interface (IBI) is proposed as a hypothesis-generating conceptual framework for organizing potential interactions among immune, cellular, extracellular, vesicular, and physicochemical processes during pathological biomineralization. Nutritional status can modulate several processes relevant to this framework, including mineral homeostasis, inflammatory and oxidative signaling, extracellular matrix regulation, and EV biology. Magnesium, vitamin K_2_, selected polyphenols, and omega-3 fatty acids provide examples of nutritional factors with varying levels of experimental and clinical evidence, particularly in vascular, renal, and inflammatory contexts. These effects should not be assumed to operate equivalently across tissues or disease states.

A critical evidence gap remains at the intersection of nutrition and tumor-associated biomineralization. Although direct tumor studies support regulated components of mineral formation, there is currently insufficient evidence to establish that defined nutritional exposures regulate the initiation, quantity, composition, maturation, or spatial distribution of tumor-associated mineral deposits. The proposed nutrition–IBI–tumor biomineralization relationship should therefore be regarded as hypothesis-generating rather than an established causal pathway.

Future studies should directly test this relationship by combining defined nutritional interventions with quantitative assessment of tumor mineralization. Integration of mineral-phase characterization, spatial molecular profiling, EV analysis, extracellular matrix assessment, and immune-cell phenotyping could determine whether nutrient-associated biological changes occur within the same microenvironments as mineral formation. Comparative studies of vascular and tumor models will also be important for distinguishing genuinely shared mechanisms from tissue-specific processes.

As a narrative review, this article integrates evidence across nutrition, vascular calcification, immunology, EV biology, mineral metabolism, and tumor-associated biomineralization rather than providing an exhaustive systematic assessment. Heterogeneity in experimental systems, nutritional exposures, disease models, and outcome measures limits direct comparison across studies. Most importantly, the scarcity of studies directly examining nutritional exposure and tumor mineralization within the same experimental framework currently limits causal inference.

Overall, the available evidence supports the IBI as a conceptual framework for organizing established mechanisms and defining experimentally testable relationships, rather than as a validated causal model. Its principal value is to identify where established nutritional and vascular mechanisms intersect with emerging evidence from tumor-associated biomineralization and, equally importantly, where evidence remains absent. Determining whether nutritional modulation meaningfully influences tumor-associated mineralization will require direct experimental investigation rather than extrapolation from vascular calcification.

## Figures and Tables

**Figure 1:**
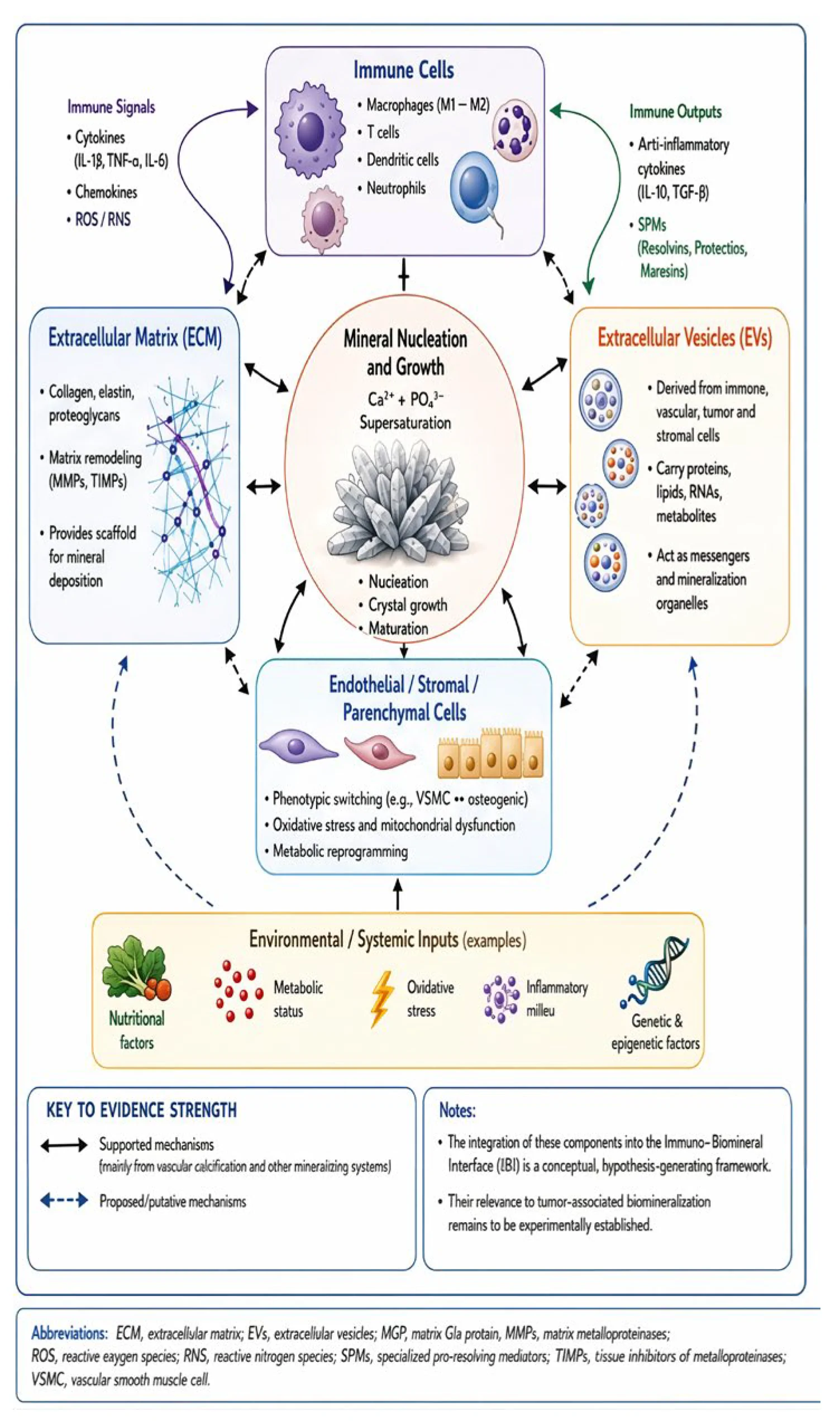
The proposed Immuno–Biomineral Interface (IBI): conceptual framework of pathological biomineralization. The IBI describes coordinated interactions among immune cells, extracellular vesicles (EVs), extracellular matrix (ECM), endothelial/stromal/parenchymal cells, and mineral precursors within pathological mineralizing microenvironments. Immune signaling, oxidative stress, cellular phenotypic changes, ECM remodeling, and EV-mediated communication may influence local calcium–phosphate homeostasis, mineral nucleation, crystal growth, and maturation. These mechanisms are supported predominantly by evidence from vascular calcification and other mineralizing systems. Black dashed arrows indicate proposed or putative mechanistic interactions that require further validation. Their integration into the IBI represents a conceptual, hypothesis-generating framework; the relevance and extent of these interconnected mechanisms in tumor-associated biomineralization remain to be experimentally established.

**Figure 2:**
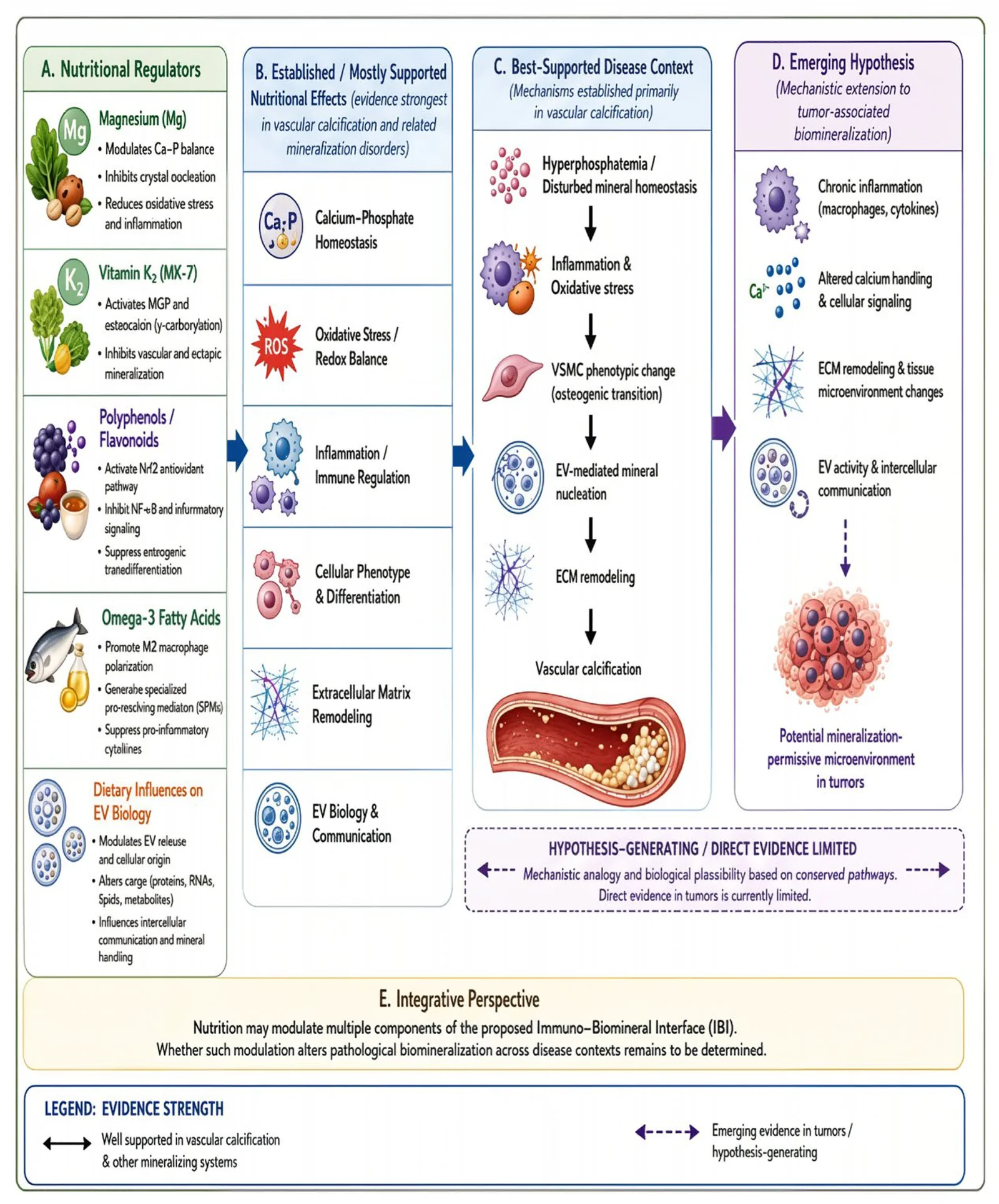
Nutritional regulation of the proposed Immuno–Biomineral Interface (IBI) and its potential extension to tumour-associated biomineralization. Nutritional regulators, including magnesium, vitamin K_2_, polyphenols/flavonoids, omega-3 fatty acids, and dietary influences on extracellular vesicle (EV) biology (A), may modulate biological processes relevant to the IBI, including calcium–phosphate homeostasis, redox balance, immune regulation, cellular phenotype and differentiation, extracellular matrix remodeling, and EV-mediated communication (B). Evidence for these processes is strongest in vascular calcification and related mineralizing disorders, where disturbed mineral homeostasis, inflammation and oxidative stress, vascular smooth muscle cell phenotypic change, EV-mediated mineral nucleation, and extracellular matrix remodeling contribute to pathological mineralization (C). Extension of these mechanisms to tumour-associated biomineralization represents an emerging, hypothesis-generating concept (D). Direct evidence linking nutritional modulation of the IBI to tumour-associated biomineralization is currently limited.

**Table 1:** Nutritional Factors, Biological Pathways, and Potential Relevance to the Immuno–Biomineral Interface.

Nutritional Factor	Primary Molecular Targets/Pathways	Potential Effects on the Immuno–Biomineral Interface	Evidence in Vascular Calcification	Potential Relevance to Tumor-Associated Biomineralization
Magnesium	Calcium–phosphate crystallization; TRPM7; RUNX2; ALP; oxidative-stress pathways	May influence calcium–phosphate crystallization, osteogenic signaling, oxidative stress, and inflammatory responses	Substantial experimental evidence; clinical and epidemiological evidence is more variable	Hypothesis-generating: may influence mineral nucleation and inflammatory or matrix-remodeling processes in tumor microenvironments; direct evidence is limited
Vitamin K_2_	Matrix Gla protein (MGP); γ-carboxylation pathways; osteocalcin-associated pathways	Supports activation of vitamin K-dependent proteins involved in mineralization control and extracellular-matrix homeostasis	Mechanistic and clinical evidence supports a role in calcification-related pathways, although intervention evidence remains heterogeneous	Hypothesis-generating: may influence extracellular-matrix mineralization and calcification-inhibitory pathways in tumors; direct evidence is very limited
Polyphenols and selected flavonoids	Nrf2; NF-κB; PI3K/AKT; ERK; SIRT1; RUNX2	Can modulate oxidative stress, inflammatory signaling, cellular differentiation, and macrophage-associated responses	Extensive experimental evidence; clinical evidence is heterogeneous and compound-specific	Hypothesis-generating: individual compounds may influence inflammatory and oxidative environments associated with mineralization; direct evidence in tumor-associated calcification remains limited
Omega-3 fatty acids	AMPK/SIRT1; PPAR-γ; NF-κB; specialized pro-resolving mediators	Can promote resolution of inflammation and modulate macrophage responses, oxidative stress, and inflammatory signaling	Substantial experimental and clinical evidence for anti-inflammatory effects; effects on vascular calcification remain context-dependent	Hypothesis-generating: may influence tumor-associated inflammatory and immune environments that could affect mineralization; direct evidence is limited
Dietary regulation of extracellular vesicles (EVs)	EV biogenesis; cargo loading; lipid, protein, and RNA signaling; intercellular communication	Nutritional status may alter EV abundance and molecular cargo, thereby modifying inflammatory communication and potentially mineral-associated processes	Emerging mechanistic evidence linking EV biology with nutritional status and vascular mineralization	Hypothesis-generating: nutritional modulation of EV-mediated mineral nucleation and tumor–immune communication remains largely unvalidated

**Table 2:** Biological Processes Potentially Influenced by Nutrition within the Immuno–Biomineral Interface.

Biological Process	Major Mediators	Nutritional Factors Studied	Evidence-Supported Biological Relevance	Evidence Context
Calcium–phosphate homeostasis	PTH, FGF23, vitamin D, calcium-sensing pathways	Calcium, phosphate, magnesium, vitamin D	Regulation of systemic mineral balance and availability	Established physiological mechanisms
Mineral nucleation and crystal growth	Matrix vesicles, alkaline phosphatase, hydroxyapatite-associated processes	Magnesium, vitamin K	Modulation of calcium–phosphate crystallization and endogenous calcification-inhibitory pathways	Established/emerging; context-dependent
Oxidative stress and cellular injury	ROS, Nrf2, mitochondrial signaling	Polyphenols, selected flavonoids, omega-3 fatty acids, magnesium	Modulation of oxidative and cellular stress pathways relevant to tissue injury and calcification	Supported, but compound- and context-dependent
Immune and inflammatory regulation	Macrophages, NF-κB, NLRP3 inflammasome, inflammatory mediators	Omega-3 fatty acids, selected polyphenols, magnesium	Modulation of inflammatory signaling and immune-cell phenotypes associated with chronic tissue remodeling	Supported/emerging
Extracellular matrix remodeling	Collagen, MMPs, matrix-associated proteins	Vitamin K, selected polyphenols	Modulation of matrix protein biology and tissue remodeling pathways relevant to calcification	Emerging/context-dependent
Extracellular vesicle biology	EV abundance, cargo, membrane-associated proteins, intercellular signaling	Dietary lipids, omega-3 fatty acids, selected polyphenols	Changes in EV abundance or molecular composition and associated intercellular signaling	Emerging
Pathological biomineralization	Calcium/phosphate availability, mineral nucleators, EVs, matrix components, inflammatory signaling	Multiple nutritional factors potentially acting through the pathways above	Potential modification of tissue environments that are permissive or restrictive to mineral deposition	Hypothesis-generating, particularly for tumors

**Table 3:** Knowledge Gaps and Future Research Priorities.

Current Knowledge	Remaining Gap	Recommended Future Approach
Nutritional factors influence several pathways implicated in vascular calcification, including mineral metabolism, inflammation, oxidative stress, and matrix remodeling.	The effects of defined nutritional exposures on tumor-associated biomineralization remain poorly characterized.	Comparative vascular–tumor studies using defined nutritional interventions and matched mineralization endpoints.
EVs can contribute to mineral nucleation in specific calcifying tissues.	Whether nutritional status alters the abundance, cargo, or mineralizing properties of EVs remains incompletely understood, particularly in tumors.	EV profiling combined with proteomics, lipidomics, and RNA sequencing following defined nutritional interventions.
Macrophages and inflammatory signaling participate in vascular calcification and tissue remodeling.	Nutrient-dependent interactions between immune-cell states and mineral deposition are poorly defined in tumor microenvironments.	Single-cell and spatial transcriptomic analyses coupled with immune phenotyping and quantitative mineral mapping.
Mineral composition and physicochemical properties can influence cellular and tissue responses.	The relationship between mineral phase, local immune activation, and tissue remodeling in tumor-associated biomineralization remains unclear.	Raman spectroscopy, FTIR, SEM–EDS, and other high-resolution mineral characterization approaches integrated with spatial biological profiling.
AI-assisted digital pathology can facilitate detection and spatial quantification of pathological calcification.	AI-based mineral detection has not yet been systematically integrated with nutritional exposure, molecular profiling, and biomineralization phenotypes.	AI-assisted digital pathology linked with spatial molecular data, mineral characterization, and quantitative nutritional intervention studies.
Nutritional modulation of vascular calcification has been investigated across experimental and clinical settings, although effects vary by nutrient, dose, and biological context.	Causal relationships between specific dietary factors and pathological mineralization remain incompletely established, particularly beyond the vascular system.	Controlled nutritional intervention studies with longitudinal assessment of mineralization, inflammatory status, EV biology, and tissue-specific outcomes.

## Data Availability

No new datasets were generated or analyzed during the current study. Data sharing is not applicable to this article because it is based on the review and synthesis of previously published literature.
